# Companion Dog Foster Caregiver Program for Older Veterans at the VA Maryland Health Care System: A Feasibility Study

**DOI:** 10.3390/ijerph16214285

**Published:** 2019-11-04

**Authors:** Heidi K. Ortmeyer, Lynda C. Robey

**Affiliations:** 1 Geriatric Research Education Clinical Center, VA Maryland Health Care System, Baltimore, MD 21201, USA; 2 Department of Medicine, University of Maryland School of Medicine, Baltimore, MD 21201, USA; Lynda.Robey@va.gov

**Keywords:** companion dog, veteran, physical activity, accelerometry, heart rate variability

## Abstract

Veterans experience mental health conditions at a disproportionate rate compared to their civilian counterparts, and approximately 60% of older veterans who receive their care through the United States Department of Veterans Affairs (VA) do not meet physical activity (PA) recommendations. We tested the Veterans as Foster Ambassadors program at the VA Maryland Health Care System to examine whether fostering a companion dog would improve PA and function, heart rate variability (HRV), balance, and quality of life (QOL) in older veterans. Participants wore an accelerometer for ≥10 days during each phase (30 day baseline vs. 60 day foster period) to measure daily PA (*n* = 4). Six-minute walk (6MW) and balance testing (*n* = 4) and 24 h heart rate (HR) and HRV (*n* = 2) were determined at baseline and during the foster period. Compared to baseline, there were significant increases in (a) distance during the 6MW, (b) daily steps, and (c) time spent in moderate activity during the foster period. 24 h HR decreased and time- and frequency-domain measures of HRV significantly increased in a veteran with post-traumatic stress disorder during the foster period compared to baseline. All veterans offered positive feedback about the program and indicated that it was beneficial to them. The results from this pilot study provide evidence that fostering a companion dog can improve PA, health, and QOL in older veterans. Future research conducted with a larger sample size to validate the results is warranted.

## 1. Introduction

Approximately 28% of adults ≥50 years of age report no physical activity outside of work [1]. Based on surveys from the 2003 Behavioral Risk Factor Surveillance System, 60% of veterans in this age category who receive care through the United States Department of Veterans Affairs (VA) do not meet physical activity recommendations [2]. Veterans also experience mental health conditions at higher rates compared to their civilian counterparts, with health-specific issues associated with different wartime eras [3]. For example, veterans who served in Vietnam are more likely to report lifetime depression and current mental distress compared to nonveterans matched for age (analyses controlled for gender, race, marital status, education level, income level, body mass index (BMI), and smoking and drinking status) [4]. Vietnam and Persian Gulf veterans are more often diagnosed with substance abuse disorders compared to veterans of the Iraq/Afghanistan war [5].

Dog ownership is often associated with daily exercise that meets physical activity guidelines, even in older adults. In studies utilizing activity monitors, adults ≥55 years of age walked their dogs for an average of 30 min per day (moderate-vigorous physical activity) [6] and adults ≥65 years of age spent more time walking at a moderate cadence compared to nonowners [7]. Older dog owners (>50 years) were shown to have lower BMI, fewer medical diagnoses, and used fewer prescription medications compared to age-matched nonowners [8]; activity was not taken into consideration. A meta-analysis study in older adults (>50 years) provided evidence that dog walking, and not dog ownership per se, is the mechanism by which dog ownership promotes health, including lower BMI, fewer activities of daily living limitations, and fewer doctor visits [9]. The 24 h heart rate variability was higher in pet owners compared to nonowners [10,11]. The degree to which differences in 24 h heart rate variability (HRV) between dog owners and nonowners are affected by physical activity or companionship or both (potential synergistic effect) has not been established.

The effects of companion dogs on health and activity levels in older veterans specifically are not well documented. Middle-aged and older veterans with post-traumatic stress disorder (PTSD) reported feeling calmer, less lonely, less depressed, and less worried about their family’s safety after adopting their dog [12]. Twenty out of 30 participants reported walking their dog for an average of 26 min per day [12]. Similarly, middle-aged and older veterans with HIV/AIDS reported that dog ownership enhanced physical activity, companionship, responsibility, and reduced stress [13].

The purpose of this pilot study was multi-faceted. We aimed to design and provide a companion dog foster program for older veterans with mental health conditions who received their care through the VA Maryland Health Care System (VAMHCS), which might serve as a model pilot program for other facilities working with older veterans. The pilot research study, Veterans as Foster Ambassadors, consisted of a one-month baseline period and a two-month foster period. An additional aim included helping dogs in need of a temporary home in Baltimore and surrounding areas by providing foster care and by sharing information on the benefits of fostering to veterans and health care providers. This study aims to provide evidence for a companion dog foster program as a significant, measurable intervention for older veterans.

## 2. Materials and Methods

Study procedures were approved by the University of Maryland Institutional Research Board (HP-00074763) and Institutional Animal Care and Use Committee (HKO-061701A), and the Veterans Affairs Research & Development Committee (1200930); all participants signed informed consent. Veterans were referred to the Veterans as Foster Ambassadors program by VA employees (e.g., health service psychologists) who were familiar with the veterans’ lifestyles and limitations. Veterans were eligible for the 3 month study if they met the following criteria: (a) ≥50 years of age, (b) did not currently have a companion animal living in their home, (b) did not have children under the age of 12 living in their home, (c) were not in a structured exercise program, (d) were not cognitively impaired, (e) were able to properly care for and safely exercise a dog, and (f) received care through the VAMHCS. Medical diagnoses and medications were reviewed in the VA electronic record (CPRS). We determined whether the veterans were suitable candidates to foster a rescue dog as part of the standard screening procedures employed by the rescue groups. Participants were asked to fill out a 19 question foster application (Appendix A) and receive a home visit. The principal investigator and veteran worked closely with rescue groups to find a suitable foster dog for the program. The participants were informed that (a) they could adopt their foster dog after the 3 month study period was completed (no adoption fee), (b) they would be financially responsible for the dog if they chose to adopt, and (c) the participating rescue groups would take their dogs back into the rescue group if for any reason the adopter could no longer care for the dog (for the life of the dog). Detailed information regarding the dog inclusion criteria, matching process, and veteran–dog dyad experiences is provided in Appendix B. All veterinary expenses, preventive medicine, dog food, and supplies (crate, leash, collar, harness, etc.) were provided during the foster period. All veterinary appointments during the research study were arranged and carried out by the principal investigator. Dog trainers were available for the participants throughout the entire research period as needed (e.g., leash walking, house training, sit and down commands, supervised separation). The rescue dogs did not receive specific training as emotional support dogs.

At a baseline visit at the Baltimore VA Medical Center, participants completed questionnaires, physical functioning (six minute walk, 6MW [14]) and balance (four square step test, FSST [15]) tests, and blood pressure and heart rate measures were taken. The baseline quality of life questionnaires included the Positive and Negative Affect Schedule, PANAS [16]; The Center for Epidemiologic Studies Depression Scale, CES-D [17]; the Perceived Stress Scale, PSS [18]; and the Short Form (36) Health Survey, SF-36 [19]. The participants wore an ActiGraph GT9X Link monitor (ActiGraph, Pensacola, FL) on their waist during the 6MW to measure steps (100 Hz sample rate). All baseline tests and measures (except the questionnaires) were repeated after 24–30 days at a follow-up visit. After the initial visit, the participants were sent home with an ActiGraph GT9X Link monitor (sample rate 30 Hz) to be worn on the wrist for 24 h for 7–10 days. The monitor was set to display only time and battery level (no activity measures were visible).

The participant continued to wear the monitor throughout the two-month foster period. The physical functioning and balance tests were repeated after 30 and 60 days. The mental health and quality of life questionnaires were repeated at the end of the foster period in addition to the Dog-Owner-Specific Quality of Life Questionnaire (DOQOLQ) [20], which had been modified for foster caregivers by permission of the authors. When feasible, the foster dog was fitted with a PetPace Collar (PetPace, Burlington, MA) to monitor activity, pulse, respiration, and heart rate variability (VVTI) as described previously [21] and a GPS tracker (Whistle, Mars Inc., San Francisco, CA, USA) in the event the dog needed to be located. The principal investigator visited the participant’s home at least two times per week throughout the baseline and intervention periods to (a) attach ECG and thigh-worn accelerometers, (b) remove monitors after the 24 h recording period, and (c) provide and replace wrist-worn accelerometers as needed. During the intervention period, the veterans shared their experiences and whether anything needed to be addressed (e.g., continued veterinary care was required for three of the dogs). We tested the feasibility and compliance of the Polar H7 chest strap (Polar, Bethpage, NY) and the Actiwave Cardio and ActiHeart 5 ECG monitors (camntech, Boerne, TX). For the Polar H7 monitor, the heart rate and interbeat interval (IBI, RR) data were collected via Bluetooth on the ActiGraph Link monitor at the maximum rate of 100 Hz for 5 min per day (10 days per period) while the participant was sitting and within 30 min of waking. The ECG monitors were attached directly to standard ECG electrodes, negating the need for a chest strap. The ECG data were collected at the rate of 200 Hz (Cardio) and 512 Hz (ActiHeart) 24 h per day (2–4 days per period). The accelerometers were set to 25 Hz and 100 Hz, respectively. To monitor position (lying, sitting, standing), an Actigraph was worn on the thigh concurrently with the ECG monitor.

ActiGraph data from the wrist-worn monitor (60 s epoch) and the thigh-worn monitor (1 s epoch) were downloaded using the Low-Frequency Extension [22] option and screened for wear time using ActiLife v6.13.4 software (ActiGraph, Pensacola, FL). Data were used only when the monitor was worn for a full 24 h day. Cut points were set at sedentary (<100 cpm), light (100–1951 cpm), moderate (1952–5724 cpm), vigorous (5725–9498 cpm), and very vigorous (≥9499 cpm) [23]. The position data from the thigh-worn monitor (sitting/lying, standing, stepping) were combined with the position data from the ActiHeart 5 monitor (1-s epoch) (lying, resting, active) to determine position per second.

Kubios HRV Premium v 3.3.1 software was used to analyze RR data generated from the Polar H7 monitor and ECG data generated from the ECG monitors [24]. The automatic artifact correction algorithm was applied to all data [25]. The 1440 HR and HRV results generated from the 24-h ECG were compared to heart rate (HR) and HRV data after all artifacts were removed to ensure the accuracy and reliability of 24 h results. The definitions for the HRV time- and frequency-domain parameters included in the results section are shown in Table 1.

Group baseline versus foster period physical function and activity and balance measures were compared using a paired *t*-test (2-tailed probability). Individual baseline versus foster average activity and HRV were tested using a 2 sample *t*-test (2-tailed probability). Pearson correlations were used to assess relationships between baseline and change (foster minus baseline) in questionnaire responses (two-tailed probability). All data are presented as mean ± SD, with statistical significance set at *p* < 0.05.

## 3. Results

Nine veterans were referred to the program (seven through health service psychologists), and of these, five consented to be in the research study. The other four veterans were eligible for the research study but chose not to participate. No veterans who were referred to the program were denied participation by the principal investigator.

Four of the five veterans completed the three-month research study; one dropped out before the one-month foster period was complete due to an episode of debilitating clinical depression. Only the results from the four participants who completed the entire 3-month study are presented.

The subjects had several medical comorbidities. The medical and mental health conditions of the participants included impaired glucose tolerance, diabetes, chronic pain, hypertension, hyperlipidemia, cancer, neuromuscular disorder, PTSD, substance abuse, depression, and anxiety disorder.

The participants (1 female, 3 male; 2 African American, 2 Caucasian) ranged in age from 53 to 74 years of age and in BMI from 23 to 32 kg/m^2^. Two veterans served in the Vietnam War, one post-Vietnam, and one in the Persian Gulf. Two of the participants had owned dogs in the past; two had not. Two participants chose mixed bully breed dogs (altered male, 45–70 pounds, 1–4 years of age), and two participants chose smaller-breed dogs (altered female, 12–25 pounds, 8–14 years of age). Three of the participants adopted their foster dogs after the 2 month foster period was completed. One participant had to sell his home and move into a residence that did not allow dogs; his foster dog was successfully adopted into a new family.

There were no significant changes in heart rate, systolic or diastolic blood pressure, or in body weight (all measured during the clinic visit) during the 2 month foster period compared to the 1 month baseline (no dog).

The differences between the end of the study and the baseline for positive (r = −0.98, *p* = 0.02) and negative affect (r = −0.91, *p* = 0.09) (PANAS) were inversely related to baseline values such that the participants with the least positive and greatest negative scores at baseline had the greatest increase in positive and greatest decrease in negative affects following the foster period. The same pattern was noted for depression (CES-D) (r = −0.99, *p* = 0.01) and stress (PSS) (r = −0.94, *p* = 0.06). The veteran with PTSD had the greatest increase in positive affect (+19), the greatest decrease in negative affect (−20), greatest decrease in depression (−20) and greatest decrease in stress (−17) following the 2 month foster period. There were no significant patterns noted for the SF-36 responses.

The positive and negative aspects of fostering a companion dog were captured with a modified DOQOL questionnaire [20]. The scale is 1–7; 1 = strongly disagree and 7 = strongly agree. The results are shown in Table 2.

**Figure 1:**
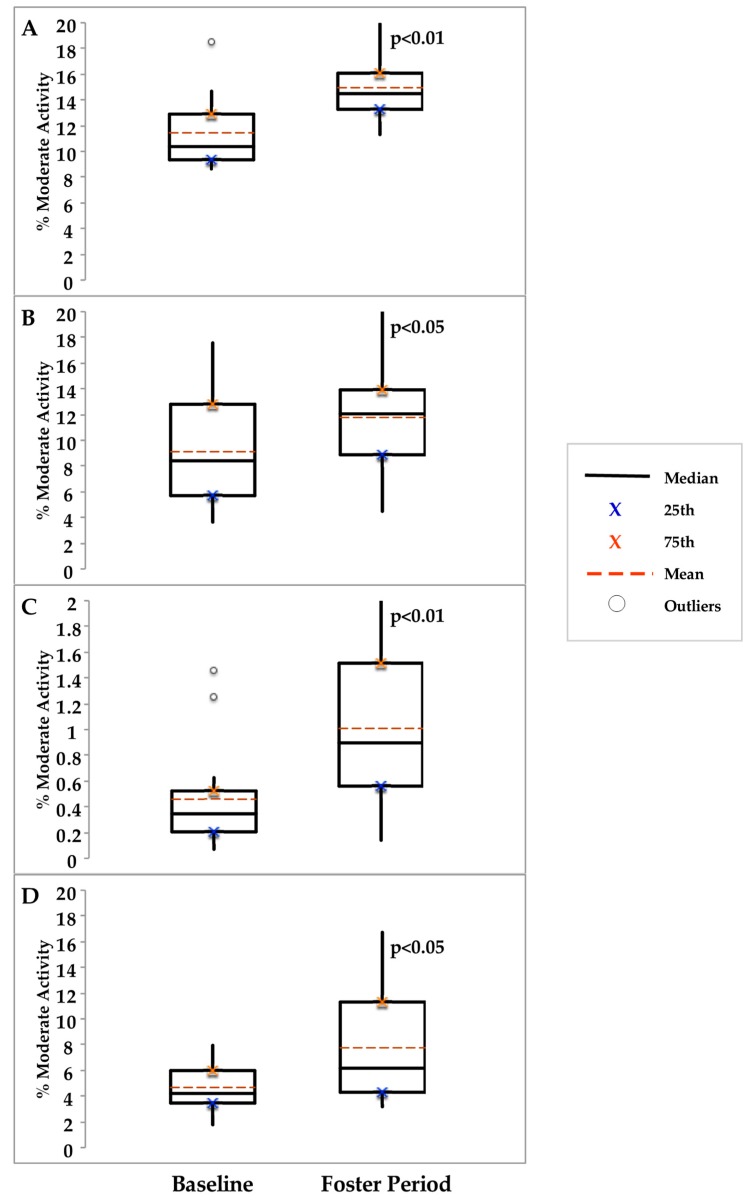
Percent time spent in moderate physical activity before (baseline) and during the foster period. Note: the scale for panel (**C**) units (Y-axis) is different than panels (**A**,**B**,**D**).

Distance and steps during the 6MW increased by 11 ± 7% (6%–22%, no dog vs. foster *p* < 0.05, *n* = 4) and 6 ± 2% (3%–8%, *p* = 0.01, *n* = 4), respectively. The time to complete the FSST decreased (not significantly) by 16 ± 13% (6%–36% decrease, *p* = 0.11, *n* = 4). Notably, one participant used knee braces during the baseline period but was able to function without them before the 1st 6MW and FSST during the foster period. Another participant used an assistive device throughout the 3 month period. Time spent in moderate activity over a 24 h period increased by 57 ± 42% (30%–119%, *p* < 0.05, *n* = 4). The individual box plots for the four participants are shown in Figure 1.

Three participants were asked to wear the Polar H7 chest strap. Participant A reported that wearing the chest strap was stressful. Participant B was compliant and wore the monitor as instructed (results shown in Table 3). Participant C reported that it was difficult to remember to put on the chest strap within 30 min of waking. Participant C switched to an ECG monitor during the baseline period. Participant D wore an ECG monitor.

Based on the combined position data from the ActiGraph (thigh) and ActiHeart (chest) monitors, participant D spent 15% less time lying (*p* < 0.05), 22% more time sitting (*p* < 0.05), 12% more time standing (*p* < 0.01), and 33% more time active (*p* < 0.05) during the foster period compared to baseline.

The veterans were asked about their experience with the program by a person not involved in the research study. Their responses are provided in Table 4.

## 4. Discussion

The purpose of the current study was to design and test a two-month companion dog foster caretaker program for older veterans with physical and mental health conditions, which might serve as a model pilot program for other facilities working with comparable veterans. We demonstrated that in close partnership with local rescue groups, it is feasible to implement a companion dog foster program for older veterans who have multiple physical and mental health conditions. This intervention was labor intensive and relied extensively on the resources of dedicated and experienced rescue group volunteers, dog trainers, and veterinary personnel. Although the sample size was small, preliminary results show improvements in physical activity and function in all four veterans, an increase in HRV in a veteran with PTSD, improvements in quality of life, and anecdotal reports of overall well-being. The attrition rate for the study (20%) was reasonable given the physical and mental health conditions of the participants.

The mechanisms by which companion dogs improve cardiovascular and mental health include increased physical activity and the powerful connection between humans and animals, as reviewed by Schreiner [26]. Differences in physical activity between dog owners and nonowners are relatively easy to measure, whereas the biological and physiological benefits of the human–animal bond itself are more difficult to capture objectively. In the current study, all four participants, no matter their baseline activity and function, significantly increased the distance walked during a six-minute walk and time spent in moderate activity after having a companion dog in their home for two months. The veteran with PTSD had the highest baseline stress, anxiety, and depression scores and the greatest improvements following the two-month foster period. This veteran also had a significant decrease in 24 h HR and significant increase in 24 h HRV following the foster period. These results are noteworthy as reduced HRV is associated with PTSD in veterans [27] and in active-duty Marines [28]. There is a growing body of literature on the importance and validity of using HRV as a noninvasive biomarker to access cardiac autonomic variation and stress as well as cognitive, emotional, social, and mental health in humans [29,30]. Measures of HRV over 24 h in conjunction with physical activity and position will provide useful tools to tease apart the influence increased activity vs. the human–animal bond (companionship) has on improvements in biological, physiological, and mental health in dog owners and caretakers.

The notion of partnering veterans who receive their care through the VA with shelter dogs is not new. The original Pets for Vets was started by Russell Lemle, Chief Psychologist at the San Francisco VA Health Care System in 2000 [31]. Dr. Lemle was acutely aware of the potential benefits a companion pet could bring to many of the veterans in his care. The non-research program provided vouchers (adoption fee and dog license fee) to any veteran seeking to adopt a dog or cat from the local shelter. In the first 11 years of the program, 169 vouchers were given out. As expected, many veterans reported a positive impact of the program in their lives; however, there were important lessons learned along the way. Mainly, numerous veterans who adopted a pet through the shelter were not able to afford their veterinary care or were not allowed to have a pet in their home, so pets were returned to the shelter. We have addressed this issue in our research study by having an advocate for the veteran work with the rescue groups to find the best-matched dog based on the home environment and based on potential financial, physical, and mental health limitations. Although not the focus of the current feasibility study, we strongly agree with the conclusion drawn by Schreiner that efforts should be made to discover ways to make companion pets more available to those with financial or housing limitations [26]. By presenting the pilot program as a foster caregiver versus an adoption program, we aimed to limit the stress the veteran might feel if she/he thought they had to make a commitment to adopt the dog. The principal investigator and rescue groups will continue to provide advice and guidance as needed for the life of the dogs. The value of working closely with responsible rescue groups cannot be overemphasized.

The veterans provided valuable feedback regarding the study design. The participants preferred to wear the ECG monitor for 24 h via two electrodes over wearing the chest strap while sitting for 5 min day within 30 min of waking. Measures of HRV over 24 h are the “gold standard” and best represent processes like circadian rhythms and the cardiovascular system’s response to a wide range of environment stimuli and workloads [32]. Shorter 5 min epochs can easily be analyzed within the 24 h timeframe and controlled by position (lying, sitting, standing) using a thigh accelerometer in conjunction with a chest accelerometer. Additionally, time spent in the various positions over a 24 h period can easily be computed when using two accelerometers.

The Dog-Owner-Specific Quality of Life Questionnaire [20], modified for foster caretakers, was used to measure the relationship between the veteran–dog dyad after the two-month foster period. Not surprisingly, all four participants strongly agreed that fostering a dog improved their level of physical activity, which is corroborated by the increase in time spent in moderate activity as determined by accelerometry. All four veterans strongly agreed that fostering a dog provided them love and affection, echoing their statements provided during the interview. Three veterans strongly agreed, and one mostly agreed that fostering provided companionship, which was a main purpose of this feasibility study. All four participants strongly disagreed that fostering a dog increased their level of stress. In future studies, responses from this and other quality of life questionnaires included in this study could be compared to changes in HRV, a quantitative measure of physiological health and stress. The time spent between the veteran and principal investigator and rescue group volunteers during the baseline and foster periods should be accounted for as these visits may have potentially influenced some of the responses on the quality of life questionnaires.

Limitations of the current study include the small sample size and lack of a control group. A larger sample size will allow for an exploration of the impact of the multiple physical and mental health conditions in older veterans who receive their care through the VA on their responses to the intervention. Another limitation is the lack of follow-up to determine whether the benefits seen over a two-month period are maintained over a longer period in those veterans who adopt their foster dogs. Future studies should be conducted with a larger sample size and for a longer time period to determine the statistical significance of a foster dog program on physiological and psychological endpoints.

## 5. Conclusions

This pilot study demonstrated that with strong support from the dog rescue groups, a companion dog foster program can be implemented in older veterans with significant physical limitations and mental health conditions. The intervention was well-received by the participants and although the sample size was small, there were beneficial effects of the intervention on their physical and psychological health. Future studies with a larger sample size should be performed to verify and extend the results of this pilot study.

## Figures and Tables

**Table 1:** Heart rate variability measures.

**Time Domain:**
RR: time interval between successive ECG R-waves
SDNN: standard deviation of normal-to-normal RR intervals
SDNNI: mean of the standard deviation of RR intervals in 5-min segments
RMSSD: Root mean square of successive RR interval differences
**Frequency Domain:**
LF: low frequency (HRV frequency band set at 0.04–0.15 Hz
HF: high frequency (HRV frequency band set at 0.15–0.4 Hz)

**Table 2:** Modified Dog-Owner-Specific Quality of Life (DOQOL) Questionnaire responses (*n* = 4).

Fostering a Dog:	Mean ± SD
Provides me love and affection	7 ± 0
Provides me companionship when I want it	6.75 ± 0.5
Provides me emotional support	6.25 ± 1.5
Improves the amount of social activities I perform	6.25 ± 1.5
Improves my ability to do things for fun outside my home	6.5 ± 1
Improves my level of physical activity	7 ± 0
Interferes with my other household responsibilities	1 ± 0
Results in damage to my belongings or property	1.75 ± 1.5
Interferes with my ability to go on vacation or leave my house	1 ± 0
Increases my level of stress	1 ± 0

SD: standard deviation.

**Table 3:** Heart rate (HR) and HRV monitor, time periods, and results (% change above baseline).

	**Participant B**	**Participant C**	**Participant D**	
Device	Polar H7	Cardio ECG	ActiHeart ECG	
Sample rate (Hz)	100	200	512	
Measurement time (min)	5	1440	1440	
Baseline measures (n)	10	2	2	
Foster period measures (n)	10	2	4	
	**Percent Change Above Baseline**			*p*-Value *
HR (bpm)	−3%	−3%	−1%	<0.005
RR (ms)	3%	4%	2%	<0.005
SDNN (ms)	1%	10%	39%	<0.05
SDNNI (ms)	*n*/a	12%	24%	<0.05
RMSDD (ms)	2%	10%	49%	=0.05
LF (n.u.)	−3%	11%	−3%	<0.01
HF (n.u.)	6%	−4%	10%	<0.05

* Participant D, Baseline versus Foster, 2 sample *t*-test.

**Table 4:** Veterans’ Experience with the Program.

My dog wakes me up in the morning when I don’t want to wake up. He’s a great companion. I always have him with me; I love him. He’s whatever you are. If you’re very active, he will be active with you. If you’re calm and not doing much, he will sit there with, lay with you. He takes on your personality pretty much.
I had just lost my oldest son. Five days later, my wife of 50 years, I found in the house dead. After all that was done, I went through a real bad depression state, and probably on the verge of being suicidal. It’s always been just me and my wife. After 50 years, she wasn’t there to pull me out of my depression state, support me, to back me up, to always be pushing me onward, making me the man she always knew I could be. Then my dog came along, and he started pulling me forward.
I’m kind of a sedentary person, I read a lot and I watch a lot of TV. My dog is a walker; she loves her walks. I think Sunday I took her on five walks. I feel better. When I wake up, I feel fresher and I get up sooner. Before my dog I would get up and be in my pajamas, and the first thing I would do is go in the living room and turn on the TV. Now I get dressed immediately and take her outside.
Having my dog has been a blessing to me. At first, she didn’t respond to me the way I felt she should, and I was afraid that she might not be a good fit. But I refused to let her go. As we got acquainted, we go and do almost everything. She brings so much joy to my life. She gets me out of the house to walk if I want to go or not. I’m glad I was put in touch with (the principal investigator) for this program.

## Supplementary Materials

The following are available online at https://www.mdpi.com/1660-4601/16/21/4285/s1, Video V1: Reunion between Veteran and Foster Dog.

## Appendix A

**Table A1:** Foster application questions.

Can you make a commitment to foster your rescue dog for two months?
Will you (the veteran) care for the dog (e.g., feeding, take outside for bathroom breaks, grooming if necessary)? Will someone else be available to help you with these tasks?
How many ADULTS reside in your household? Please indicate the gender and age for all of them.
How many CHILDREN reside in the household? Please indicate their gender and age
If your household does not include children, please indicate whether children are regular or frequent visitors to your home.
In what type of home do you live (e.g., apartment, town home, single-family home)?
Do you own or rent your home? If you rent, do you have approval from your landlord to foster a dog? Please provide landlord’s phone number so we may contact him/her.
Please describe your yard (area, fenced, pool, etc.)
Please indicate whether any members of your household have special needs.
Does anyone living in the home smoke, and if so, do they smoke indoors?
Indicate whether you have lived with a dog in the past. If so, please comment on your experience (good, bad, etc.).
Do you have a preference for the age, gender, and/or size of the foster dog?
Will you (the veteran) agree to take daily walks with the dog? We recommend two–three walks per day between 10–20 min in length.
Please describe the areas where you will walk with your dog (busy street, quiet street, path, etc.)
How much time will your foster dog spend alone during the week and on the weekend?
Do you agree to an initial visit to your home?
Please provide the names, phone numbers, and e-mail addresses of two references (not family members).
I understand that if I am approved to foster a dog through the Vets Foster Pets program I must fully read and sign the “Foster Contract”, which is a separate document from this application, and that this “Foster Contract” is a legal contract between Vets Foster Pets rescues and a foster dog caregiver, and that this agreement must be signed before I am able to accept a foster dog for care into my home.
I certify that the information entered on this application is true.

## Appendix B

Dog criteria and matching process. Adult (>1 year of age) altered (neutered/spayed) dogs that were currently being fostered in a rescue group or housed in a shelter were eligible for this study. The principal investigator (PI) showed the participants how to navigate the Petfinder website to locate rescue dogs that met their criteria (e.g., gender, size, breed). Once a dog was identified, the PI arranged a meet-and-greet through the rescue group and participated in the meeting with the veteran. The next step was to meet with a dog trainer and the rescue group to ascertain training needs and discuss the importance of daily exercise, nutrition, and care (e.g., preventive medicine). The trainer was available to the veteran throughout the 3-month study. All veterinary visits were arranged and carried out by the PI throughout the 3-month study. All adopters received a 12-month supply of heartworm and flea-tick preventive medicine for their dog.

Veteran–dog dyad 1. The veteran identified a young male mixed bully breed dog through petfinder.com. The rescue group was not able to house (foster) the dog for the 30 day baseline period. Through the dog trainer’s rescue network, we were able to find a couple to foster the dog for 30 days before the veteran–dog foster period started. Throughout the 30 day baseline period, the dog received training (e.g., house training), and the trainer was available to the veteran throughout the foster period. The dog required limited vetting (vaccines) during the baseline period. The veteran, who had not previously owned a dog, adopted the dog after the study completed.

Veteran–dog dyad 2. The veteran asked the PI to find a “big” dog as he had owned large dogs in the past. The veteran and PI visited a participating rescue group that specialized in larger breeds. At this meet-and-greet, the veteran chose a large male adult mixed bully breed dog. The dog stayed with the rescue group during the 30 day baseline period. During this time the veteran, PI, rescue group, and trainer met several times to go over training and nutrition tips. This dog suffered from long-term (>3 years) untreated allergies and required extensive veterinary care, which we started immediately after the meet-and-greet and continued throughout the study. The PI arranged and completed all veterinary visits, and all veterinary care was paid by the award from Maddie’s Fund. The veteran desired to adopt the dog after the study completed, but unfortunately had to move into a residence that would not allow dogs. A new family adopted the dog; the veteran has since visited with his foster dog and is welcome to visit in the future (see Reunion between Veteran and Foster Dog video in Supplemental Materials).

Veteran–dog dyad 3. The veteran had owned several dogs of a specific (small) breed in the past and asked the PI to find that breed of dog for the study. The veteran also indicated that he would like to foster a senior dog. The PI reached out to several groups that had this specific breed but could not find a group willing to participate in the study. Some of the reasons given for not allowing the veteran to foster included the fact that he lived in an apartment and didn’t have a fenced yard. The PI identified a small female senior dog of a different breed (American Eskimo) from a rescue group. The dog stayed with the PI during the baseline period. The dog required extensive follow-up vetting during the study (paid for by the rescue group). The veteran adopted the dog after the study completed.

Veteran–dog dyad 4. The veteran had not owned a dog before and was not sure what type of dog would be a good fit. The PI and veteran visited a rescue group and met several available dogs. The veteran chose an older small female dog (Yorkshire Terrier) that had been recently rescued as a stray. The dog was found on the side of a highway by animal control in severe need of medical care. The dog required extensive follow-up medical attention throughout the study period (paid for by the award from Maddie’s Fund). The dog stayed with the PI during the baseline period. The veteran adopted the dog after the study completed.

A note on financial responsibility post-adoption. Every effort will be made (e.g., fund-raising efforts) by the PI and participating rescue groups to allow for the veteran to keep their adopted dog in the event the veteran experiences financial hardship and can no longer afford their care. For example, one of the adopted dogs (dyad 3) has a sponsor to cover all medical expenses for the life of the dog as needed.
